# The prognostic role of MRI-based radiomics in tongue carcinoma: a multicentric validation study

**DOI:** 10.1007/s11547-024-01859-y

**Published:** 2024-08-03

**Authors:** Marta Tagliabue, Francesca Ruju, Chiara Mossinelli, Aurora Gaeta, Sara Raimondi, Stefania Volpe, Mattia Zaffaroni, Lars Johannes Isaksson, Cristina Garibaldi, Marta Cremonesi, Anna Rapino, Susanna Chiocca, Giacomo Pietrobon, Daniela Alterio, Giuseppe Trisolini, Patrizia Morbini, Vittorio Rampinelli, Alberto Grammatica, Giuseppe Petralia, Barbara Alicja Jereczek-Fossa, Lorenzo Preda, Marco Ravanelli, Roberto Maroldi, Cesare Piazza, Marco Benazzo, Mohssen Ansarin

**Affiliations:** 1 Division of Otolaryngology and Head and Neck Surgery, European Institute of Oncology IRCCS, Via Ripamonti 435, 20141 Milan, Italy; 2 Department of Biomedical Sciences, University of Sassari, Sassari, Italy; 3 Division of Radiology, European Institute of Oncology IRCCS, Milan, Italy; 4 Department of Statistics and Quantitative Methods, University of Milan-Bicocca, Via Bicocca Degli Arcimboldi, Milan, Italy; 5 Department of Experimental Oncology, European Institute of Oncology IRCCS, Milan, Italy; 6 Division of Radiation Oncology, European Institute of Oncology, IRCCS, Milan, Italy; 7 Department of Oncology and Hemato-Oncology, University of Milan, Milan, Italy; 8 Unit of Radiation Research, IEO European Institute of Oncology, IRCCS, Milan, Italy; 9 Postgraduate School of Radiodiagnostic, University of Milan, Milan, Italy; 10 Department of Otorhinolaryngology and Skull Base Microsurgery-Neurosciences, ASST Ospedale Papa Giovanni XXIII, Bergamo, Italy; 11 Unit of Pathology, E.O. Ospedali Galliera, Genoa, Italy; 12 Unit of Otorhinolaryngology-Head and Neck Surgery, Department of Medical and Surgical Specialties, Radiological Sciences and Public Health, ASST Spedali Civili of Brescia, University of Brescia, 25123 Brescia, Italy; 13 Diagnostic Imaging and Radiotherapy Unit, Department of Clinical, Surgical, Diagnostic, and Pediatric Sciences, University of Pavia, Pavia, Italy; 14 Radiology Institute, Fondazione IRCCS Policlinico San Matteo, Pavia, Italy; 15 Department of Medical and Surgical Specialties, Radiological Sciences, and Public Health, University of Brescia, School of Medicine, Brescia, Italy; 16 Department of Otorhinolaryngology, Fondazione IRCCS Policlinico San Matteo, Pavia, Italy

**Keywords:** Radiomics, Tongue cancer, Head and neck cancer, Precision medicine, Prognosis prediction, Omics

## Abstract

**Purpose:**

Radiomics is an emerging field that utilizes quantitative features extracted from medical images to predict clinically meaningful outcomes. Validating findings is crucial to assess radiomics applicability. We aimed to validate previously published magnetic resonance imaging (MRI) radiomics models to predict oncological outcomes in oral tongue squamous cell carcinoma (OTSCC).

**Materials and methods:**

Retrospective multicentric study on OTSCC surgically treated from 2010 to 2019. All patients performed preoperative MRI, including contrast-enhanced T1-weighted (CE-T1), diffusion-weighted sequences and apparent diffusion coefficient map. We evaluated overall survival (OS), locoregional recurrence-free survival (LRRFS), cause-specific mortality (CSM). We elaborated different models based on clinical and radiomic data. C-indexes assessed the prediction accuracy of the models.

**Results:**

We collected 112 consecutive independent patients from three Italian Institutions to validate the previously published MRI radiomic models based on 79 different patients. The C-indexes for the hybrid clinical-radiomic models in the validation cohort were lower than those in the training cohort but remained > 0.5 in most cases. CE-T1 sequence provided the best fit to the models: the C-indexes obtained were 0.61, 0.59, 0.64 (pretreatment model) and 0.65, 0.69, 0.70 (posttreatment model) for OS, LRRFS and CSM, respectively.

**Conclusion:**

Our clinical-radiomic models retain a potential to predict OS, LRRFS and CSM in heterogeneous cohorts across different centers. These findings encourage further research, aimed at overcoming current limitations, due to the variability of imaging acquisition, processing and tumor volume delineation.

**Supplementary Information:**

The online version contains supplementary material available at 10.1007/s11547-024-01859-y.

## Introduction

Radiomics represents one of the most attractive fields in medicine that has taken hold in the last 10 years [1]. Radiomic features analysis from pretreatment images aimed at acquiring all possible data on cancer characteristics and prognosis prediction [2, 3]. Currently, oncological outcomes can be predicted only based on pathological and clinical tumor stage, leading to the need for innovative strategies to better foresee patients’ prognosis. Therefore, the quantitative information extracted from medical images, is the basis for radiomics to become unbiased and independent support in daily clinical practice. Despite the large number of published studies, the application of radiomics to clinical practice is not yet feasible because of the lack of radiomic validation in different cohorts [3–5].

In our study, we apply the radiomics workflow to oral tongue squamous cell carcinoma (OTSCC), the tumors that most frequently affect the oral cavity [6]. For preoperative staging, magnetic resonance imaging (MRI) is the gold standard imaging [7]. The first-choice therapeutic approach for OTSCC is surgery, and the prognosis reported in the literature remains 60% at 5 years [8, 9]. Survival ranges from 80% for early stages (I–II) to 30% for advanced stages (III–IV). There is an increasing awareness that these estimates may not adequately fit single patient's history, calling for a more tailored prediction approach. In precision medicine, accurate risk prediction is necessary to plan personalized therapeutic/follow-up schemes based on the individual survival curves, leading to the ability to predict “individual patient’s survival,” instead of a too general “global survival” [4].

To date, published studies have underlined how the hybrid clinical-radiomic predictive models are better than the clinical or radiomic models for predicting patients’ outcomes [10–12].

Validating a radiomic model should be mandatory [5, 13] to confirm its potential use in clinical settings. The validation cohort should be independent and external, based on subjects other than the training set cohort [14], as validations based on the reference model's internal cohort are generally less robust [5, 14–16].

We have recently published a study with clinical-radiomic models in patients with OTSCC capable of preoperatively predicting patients' prognosis, better than clinical models alone [10]. In this published analysis we assessed preoperative MRI of 79 patients with OTSCC, aiming to define potential prognostic biomarkers using radiomic features. Upon evaluating clinical and radiomic features, the radiomic score maintained statistical significance in almost all clinical-radiomic predictive models and apparent diffusion coefficient map (ADC) MRI provided the best fit to the models.

According to the concept set out in the previous paragraph on the importance of external validation of radiomics studies [14] to highlight stability and reproducibility, the aim of this paper is to externally validate our previously published clinical-radiomics models [10] through a different and independent cohort of patients.

## Materials and methods

### Clinical dataset

We performed a retrospective radiomic analysis on preoperative MRI of consecutive patients with OTSCC surgically treated in a multicentric setting: Surgery performed at the European Institute of Oncology (IEO) Milan, Italy with MRI performed in other Italian facilities collected in the online imaging storage (IEO EXT); Surgery and Imaging at Policlinico San Matteo, Pavia, Italy (PV); Surgery and Imaging at Spedali Civili, Brescia, Italy (BS).

The study was approved by the Ethics Committee of the IEO and by the IEO Radiomic Board (UID 2520), Spedali Civili of Brescia (120/Reg.IX) and Policlinico San Matteo of Pavia (96379/2020).

Inclusion criteria were: diagnosis of OTSCC; surgery performed between 2010 and 2019; preoperative MRI (≤ 4 weeks before surgery) including contrast-enhanced T1-weighted (CE-T1) and diffusion-weighted (DWI) sequences with at least two *b* values for the calculation of apparent diffusion coefficient map (ADC).

Exclusion criteria were: concurrent or previous cancer in head and neck (HN) region; inadequate follow-up information (irretrievable medical information data); inadequate MRI for tumor volume segmentation and quantitative analysis (i.e., cases were excluded when the primary tumor was not detectable or artifacts significantly degraded the images).

Clinical, pathological, treatment and follow-up information were collected from medical reports. All patients were re-staged according to 7th and 8th edition of AJCC TNM [17, 18]. To re-stage all patients using the AJCC 8th edition we re-evaluated and collected all histological and radiological Depth of Invasion (DOI). In both, histological specimen and radiological MRI DOI was measured perpendicularly from the line connecting the adjacent normal mucosal basement membrane to the deepest point of tumor invasion.

All patients were surgically treated and then the need of adjuvant therapy was discussed and defined within a multidisciplinary tumor board according to stage disease [19, 20].

For at least 5 years from the end of treatment, all patients underwent state-of-art clinical assessments and procedures of standardize oncological follow-up according to the NCCN guidelines [20]. The distant or local events as metastases or locoregional recurrence were recorded during the follow-up visits. Patients who did not attend scheduled follow-up appointments were phone interviewed to verify and update their medical information,

### MRI acquisition parameters, segmentation, feature extraction

For the Training Group (TG), MRIs were performed at IEO on a 1.5-T system (Magnetom Avanto, Siemens Healthineers, Erlangen, Germany) with a dedicated 16-channel receive-only radiofrequency HN coil [10]. DWI sequences were obtained via single-shot spin-echo and echo-planar imaging (field of view 250 × 250 mm, TR/TE 5000/77 ms, slice thickness 5 mm, spacing between slices 1 mm, bandwidth 1565 Hz/pixel). Three different b values were used (*b* = 0.500 and 900 s/mm^2^) with diffusion-sensitizing gradients applied in three orthogonal directions to obtain trace-weighted images [10]. ADC maps derive from a mono-exponential analysis of diffusion-weighted images. The imaging protocol included post-contrast (Gadolinium 0.2 ml/kg) isotropic T1-w images (acquisition matrix 263 × 384 mm, field of view 187 × 240 mm, TR/TE 7.43/2.88 ms, slice thickness 0.6 mm) [10].

For the Validation Group (VG) at Policlinico San Matteo of Pavia, the MRI was performed on a 1.5-T system (Aera, Siemens Healthineers, Erlangen, Germany) with the dedicated 16-channel receive-only radiofrequency HN coil. DWI sequences were obtained via single-shot spin-echo and echo-planar imaging (260 × 260 mm, TR/TE 7075 /58 ms, slice thickness 3 mm, spacing between slices 3.3 mm, bandwidth 1540 Hz/pixel). To obtain trace-weighted images, two different b values were used (*b* = 50, 800 s/mm^2^) with diffusion-sensitizing gradients applied in three orthogonal directions. The imaging protocol included post-contrast (Gadolinium 0.2 ml/kg) isotropic T1-w images (acquisition matrix 256 × 256, field of view 260 × 260 mm, TR/TE 8.23/2.39 ms, slice thickness 1 mm).

For the VG at Spedali Civili of Brescia, the MRI was performed on a 1.5-T system (Aera, Siemens Healthcare Sector, Erlangen, Germany) with the dedicated 16-channel receive-only radiofrequency HN coil. DWI sequences were obtained via single-shot spin-echo and echo-planar imaging (field of view 250 × 250 mm, TR/TE 4000/60 ms, slice thickness 3 mm, spacing between slices 1 mm, bandwidth 1447 Hz/pixel). Two different b values were used (*b* = 0 and 1000 s/mm^2^) with diffusion-sensitizing gradients applied in three orthogonal directions to obtain trace-weighted images. The imaging protocol included post-contrast (Gadolinium 0.2 ml/kg) isotropic T1-w images (acquisition matrix 448 × 350, field of view 270 × 210 mm, TR/TE 8.2/3.16 ms, slice thickness 0.6 mm).

The MRI characteristics described above cannot be described in the same detail for patients treated at IEO with imaging performed in different external centers (IEO EXT). This is because the IEO imaging storage archives only the DICOM images of the MRIs performed in the external hospitals, leading to some lacking details.

**Fig. 1:**
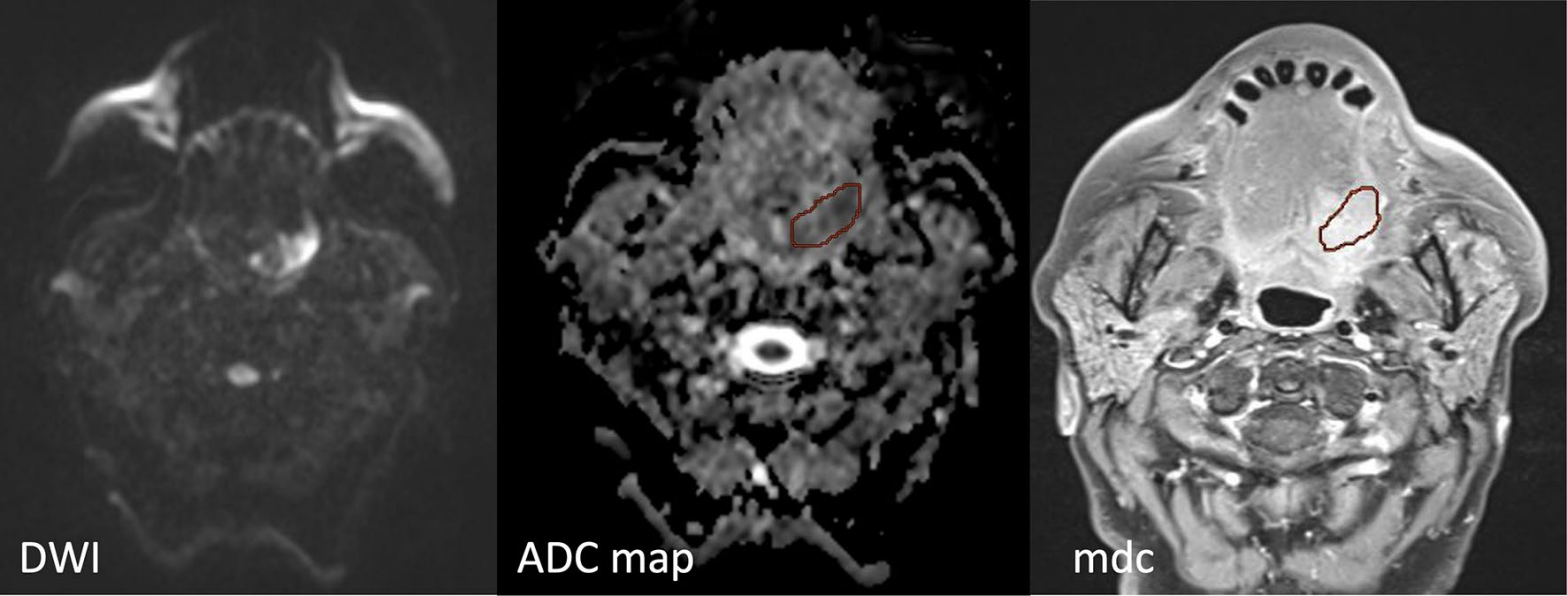
Region of interest (ROI) manually delineated on the contrast-enhanced comparing them with ADC map imaging excluding peritumoral edema. ROIs on the ADC map was based on the lesion observed in the DWI sequence

Two dedicated HN radiologists [one senior radiologist (FR) with 7 years’ experience in HN and one junior (AN) with 1 year of experience in HN] manually segmented the entire tumor volume (region of interest, ROI) in CE-T1 sequences and ADC maps (Fig. 1). Inter-reader agreement was evaluated and all discrepancies were solved through discussion. The radiologists were unaware of the patient characteristics and their follow-ups status.

DICOM files and ROIs were extracted as radiotherapy (RT) structure files on AW Server 3.2 workstation (GE Healthcare, Milwaukee, WI) and exported in DICOM format. Features extraction was performed using the Image Biomarker Standardization Initiative (IBSI) compliant tool PyRadiomics v3.0.1 (Numpy version 1.20.3, SimpleITK version 2.0.2, PyWavelet version 1.1.1) with default settings. The radiomic features were extracted from all available filter classes (Laplacian of Gaussian, wavelet, logarithm, exponential, local binary pattern 2D, local binary pattern 3D, square, square root and gradient). In total, 1967 features were extracted from each segmentation.

### Oncological outcomes endpoints

Overall survival (OS) was defined as the time from surgery until death from any cause or the last contact date if alive. Locoregional recurrence-free survival (LRRFS) was defined as the time from surgery until locoregional recurrence or the last contact date without locoregional recurrence. LRRFS includes relapses on tumor (T), lymph nodes (N) or T and N, no distant metastasis or second primary were included. Cause-specific mortality (CSM) was defined as the time from surgery until the date of death for OTSCC. In case of no death due to OTSCC, the observation was censored at the last follow-up visit or the date of death for other causes. The Kaplan–Meier was estimated for 10-year OS, LRRFS and CSM.

### Statistical analysis

We performed a validation of ahead trained models on a different and independent cohort of patients (TG) [10]. Specifically, we applied the radiomic models, the pretreatment clinical models (including gender, age and clinical status of the lymph node), and the posttreatment clinical models (including state of margins, state of lymph nodes, presence extracapsular metastases (ECE) [10].

Frequency and percentages for categorical variables and median and interquartile range (IQR) for continuous variables were used to summarize patients’ characteristics in the validation cohort. Pearson's chi-square and Fisher's exact tests were used to test for differences between categorical variables. Wilcoxon's rank sum and Kruskal–Wallis tests were used to assess for a continuous variable difference between two or more groups, respectively. First, clinical differences and median follow-up between patients in the TG and VG were analyzed, as well as between the centers involved. Differences of the radiomic features between VG and TG were evaluated (Supplementary Tables 1 and 2). If significant differences among features were identified, these features were harmonized with the COMBAT method (“EZ. combat” library in R), considering each center as a batch variable [21]. The difference in OS, LRRFS and CSM between the TG and VG was tested with the Log-rank test. The centers involved in validation were compared in the OS, LRRFS and CSM. Risk estimates were quantified by hazard ratio (HR) and 95% confidence intervals (95%-CI).

Radiomic information was aggregated in the statistical analysis within a “radiomic score,” built with the coefficient used in the previous study [10]. For each patient, the radiomic score was defined as the sum of the product between the regression coefficients and their respective feature values. We evaluated the previously presented predictive models: clinical model (pre- and posttreatment), radiomic model and clinical-radiomic model (pre- and posttreatment). The models’ accuracy was tested by using the Concordance index (C-index), a measure of goodness of fit for survival outcomes, which ranges from 0 (poor predictive model) to 1 (hypothetical perfect predictive model). All analyses were repeated for CE-T1 and ADC. Lastly, a Bootstrap analysis with 5000 repetitions was performed to determine a 95%-CI for the C-index estimate. As an exploratory analysis, we used the VG as a new dataset to build a new model with new coefficients using the preselected clinical variables and the radiomic score obtained in the training model. Analyses were performed using R (4.1.1).

## Results

The study included 112 patients as VG. TG models were built on 79 different patients [10].

Out of the 112 patients included in the study based on inclusion and exclusion criteria, after MRI quality images reviewed by the HN Radiologists, 108 patients were included in the VG for CE-T1 sequences (IEO EXT *N* = 25; PV *N* = 19; BS *N* = 64) and 83 patients for ADC from DWI sequences (IEO EXT *N* = 13; PV *N* = 7; BS *N* = 63).

Table 1 reports the clinical and pathological characteristics of patients in the two study groups: TG (79 patients), VG (112 patients) and Overall (191 patients). Sixty-nine percent of the entire population was male, with an average age of 62 (IQR: 47–70), with VG including significantly older patients (64 years, IQR: 52–73) than the TG (55 years, IQR: 41–67).

**Table 1:** Patients, tumor and treatment characteristics, in the overall cohort, in the training and validation groups

Characteristics	Overall, *N* = 191	Training, * N* = 79^1^	Validation, * N* = 112^1^	*p* value^2^
Age	62 (47, 70)	55 (41, 67)	64 (52, 73)	**0**.**005**
< 45	43 (23)	24 (30)	19 (17)	**0**.**029**
**≥ **45	148 (77)	55 (70)	93 (83)	
Sex				0.13
Female	60 (31)	20 (25)	40 (36)	
Male	131 (69)	59 (75)	72 (64)	
Tobacco smoking habits				0.9
No smoker	72 (39)	30 (38)	42 (39)	
Smoker/ex smoker	115 (61)	49 (62)	66 (61)	
Missing	4	0	4	
PACK/YEAR (on smokers)	30 (18, 40)	30 (15, 40)	30 (20, 45)	0.7
Missing (*N* patients)	76	30	46	
Alcohol intake ≥ 3 Units per day	34 (19)	16 (21)	18 (17)	0.6
Missing	11	2	9	
Radiological DOI (mm)	15 (10, 21)	16 (9, 23)	14 (11, 20)	0.6
≤ 5	6 (3.1)	4 (5.1)	2 (1.8)	0.076
5 < mm ≤ 10	46 (24)	24 (30)	22 (20)	
> 10	139 (73)	51 (65)	88 (79)	
cT (TNM VIII ed.)				**0**.**03**
cT1	4 (2.1)	3 (3.8)	1 (0.9)	
cT2	46 (24)	25 (32)	21 (19)	
cT3	125 (65)	48 (61)	77 (69)	
cT4	16 (8.4)	3 (3.8)	13 (12)	
cN (TNM VIII ed.)				0.9
cN0	102 (53)	45 (57)	57 (51)	
cN1	24 (13)	10 (13)	14 (12)	
cN2	62 (32)	23 (29)	39 (35)	
cN3	3 (1.6)	1 (1.3)	2 (1.8)	
cStage (TNM VIII ed.)				**0**.**05**
I	6 (3.1)	3 (3.8)	3 (2.7)	
II	32 (17)	20 (25)	12 (11)	
III	84 (44)	32 (41)	52 (46)	
IV	69 (36)	24 (30)	45 (40)	
Grading				0.6
G1	37 (19)	18 (23)	19 (17)	
G2	102 (54)	40 (51)	62 (55)	
G3	51 (27)	20 (26)	31 (28)	
Missing	1	1	0	
Tumor diameter (mm)	30 (22, 40)	28 (20, 42)	30 (23, 40)	0.7
≤ 20	42 (22)	21 (27)	21 (19)	0.2
20 < mm ≤ 40	101 (53)	36 (46)	65 (58)	
> 40	48 (25)	22 (28)	26 (23)	
Glossectomy type^20^				**0**.**02**
II (transoral)	26 (14)	16 (20)	10 (9)	
III–V (major en block surgery)	165 (86)	63 (80)	102 (91)	
Histological DOI (mm)		11 (9, 11)	12 (10, 18)	
≤ 5	20 (10)	12 (15)	8 (7.1)	0.12
5 < mm ≤ 10	32 (17)	10 (13)	22 (20)	
> 10	139 (73)	57 (72)	82 (73)	
Micrometastases				0.2
No	179 (94)	72 (91)	107 (96)	
Yes	12 (6.3)	7 (8.9)	5 (4.5)	
Multifocality				0.5
No	176 (92)	74 (94)	102 (91)	
Yes	15 (7.9)	5 (6.3)	10 (8.9)	
Surgical margins				**0**.**017**
Negative	156 (82)	72 (91)	84 (75)	
Positive	17 (8.9)	3 (3.8)	14 (12)	
Close (< 1 mm)	18 (9.4)	4 (5.1)	14 (12)	
Vascular invasion				**< 0**.**001**
No	141 (74)	71 (90)	70 (62)	
Yes	50 (26)	8 (10)	42 (38)	
Perineural infiltration				**< 0**.**001**
No	88 (46)	49 (62)	39 (35)	
Yes	103 (54)	30 (38)	73 (65)	
pT (TNM VIII ed.)				0.2
pT1	16 (8.4)	10 (13)	6 (5.4)	
pT2	31 (16)	10 (13)	21 (19)	
pT3	95 (50)	35 (44)	60 (54)	
pT4a	47 (25)	23 (29)	24 (21)	
pTis	2 (1.0)	1 (1.3)	1 (0.9)	
pN (TNM VIII Ed.)				**0**.**008**
pN0	67 (35)	19 (24)	48 (43)	
pN1	24 (13)	10 (13)	14 (12)	
pN2	32 (17)	16 (20)	16 (14)	
pN3	54 (28)	23 (29)	31 (28)	
pNx	14 (7.3)	11 (14)	3 (2.7)	
Stage (TNM VIII ed.)				0.08
0	1 (0.5)	0 (0)	1 (0.9)	
I	14 (7.3)	9 (11)	5 (4.5)	
II	18 (9.4)	9 (11)	9 (8.0)	
III	60 (31)	17 (22)	43 (38)	
IVA	44 (23)	21 (27)	23 (21)	
IVB	54 (28)	23 (29)	31 (28)	
Adjuvant RT	66 (35)	29 (37)	37 (33)	0.6
CT/RT adjuvant	73 (38)	26 (33)	47 (42)	0.2
No adjuvant CT/RT	52 (27)	24 (30)	28 (25)	0.4
Time of follow-up (months)	28 (15, 60)	30 (17, 65)	27 (14, 53)	0.3
AWD	65 (34)	24 (30)	41 (37)	0.4
NED	126 (66)	55 (70)	71 (63)	

Bold values indicate the *p* value < or = 0.05

^1^Median (Inter quartile range-IQR); n ()

^2^*p* values for the difference between Training and Validation cohorts: Wilcoxon rank sum or Kruskal–Wallis test for continuous variables; Pearson's Chi-squared test or Fisher's exact test for categorical variables

DOI, depth of invasion; c, clinical staging; p, pathological staging; RT, radiotherapy; CT/RT, chemoradiotherapy; AWD, alive with disease; NED, not evidence of disease

Clinical staging (c) showed differences between TG and VG in tumor (cT) classification (*p* = 0.03) and clinical Stage cTNM (*p* = 0.05) with higher stages found for patients in TG.

Postsurgical information: vascular invasion, perineural invasion, surgical margins (positive or close) and lymph node stage (pathological (p)N) were statistically different between the two groups TG and VG: < 0.001, < 0.001, 0.017, 0.008, respectively (Table 1).

No difference was found between the pTNM and postoperative treatments; *p* = 0.02 was found for the type of surgery, transoral (glossectomy type II) versus en block major surgery (glossectomies type III–V) [22].

The median follow-up time was 2.8 years IQR (1.84–5.44) for OS, 2.8 years (0.94–4.69) for LRRFS and 2.75 years (1.45–4.87) for CSM.

**Fig. 2:**
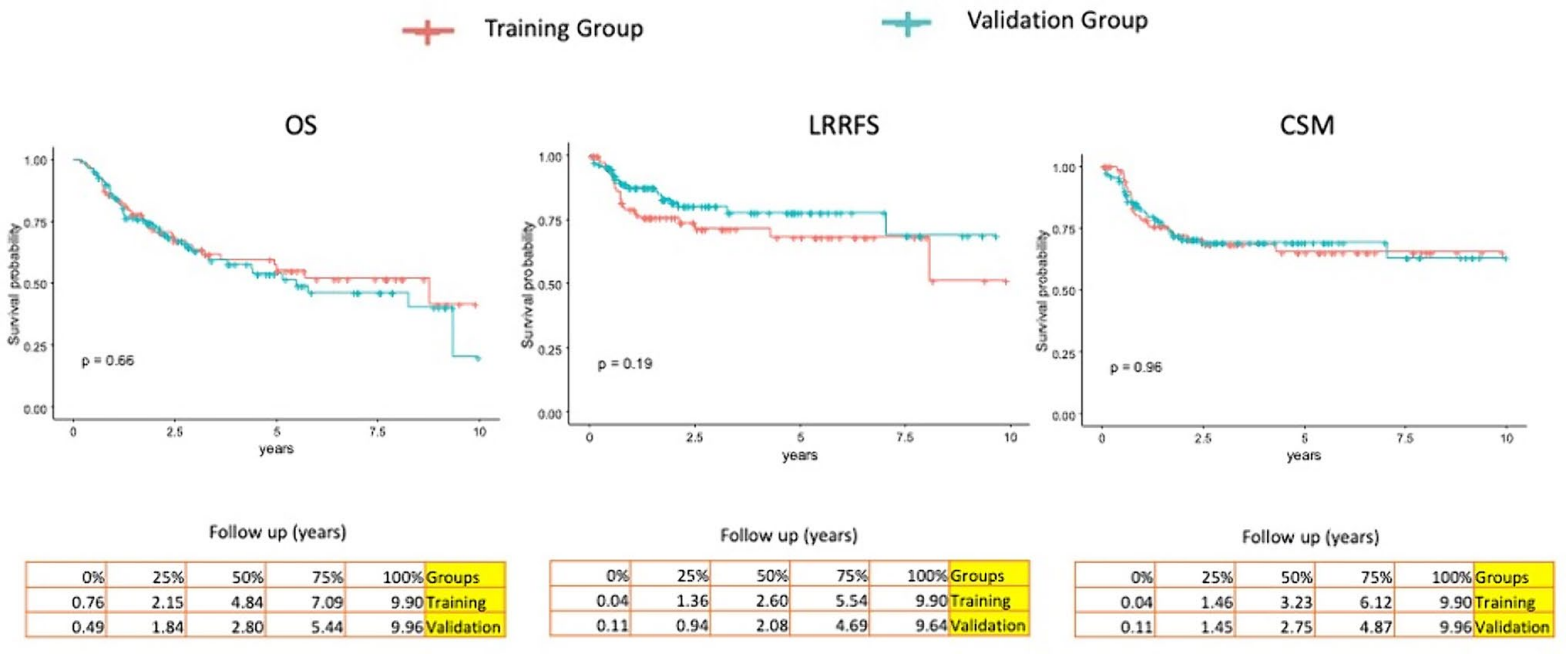
Oncological outcomes comparison between training and validation groups. OS, overall survival; LRRFS, locoregional recurrence-free survival; CSM: cause-specific mortality

No significant differences were detected between the TG and VG for OS, LRRFS and CSM (all *p* values > 0.05) (Fig. 2).

**Fig. 3:**
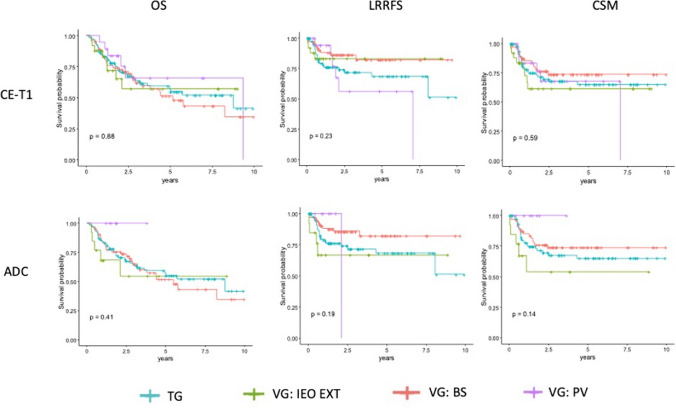
Oncological outcomes comparison between the three different clinical centers included in the Validation Group. OS, overall survival; LRRFS, locoregional recurrence-free survival; CSM, cause-specific mortality; ADC, apparent diffusion coefficient map; CE-T1, contrast enhancement T1 sequence; TG, training group; VG, validation group; IEO EXT, patient treated at IEO with MRI performed in other hospitals; BS, brescia spedali civili; PV, Pavia San Matteo

Even among the patients collected by the three different clinical centers involved in the validation, there were no differences in survival: OS, LRRFS and CSM (Fig. 3).

Features selected in our previous model for OS, LRRFS and CSM were retrieved from the VG [10]. The radiomic score was calculated on the validation dataset with the previously defined coefficient.

Tables 2 and 3 summarize all the results of the validation analysis, comparing the C-Index in the training set and the fitted C-index (“C-index-validation”) in CE-T1 and ADC map for the pretreatment and posttreatment models, respectively. Radiomic models adequately fitted each endpoint: Table 2 depicts the comparison between the C-Index in the TG and the fitted C-Index (C-Index–VG) for ADC and CE-T1 sequences in the model base on pretreatment information.

**Table 2:** Pretreatment model C-index in the training and in the validation group

Pretreatment model					
Sequence	Clinical^1^	Radiomic		Clinical-Radiomic	
		ADC	CE-T1	ADC	CE-T1
OS					
C-Index-training	0.70	0.72	0.79	0.76	0.82
	[0.57, 0.77]	[0.59, 0.78]	[0.69, 0.84]	[0.62, 0.78]	[0.73, 0.86]
C-Index-validation	ADC 0.60 [0.49, 0.70]	0.59 [0.48, 0.70]	0.59 [0.49, 0.68]	**0**.**60** [0.49, 0.71]	**0**.**61** [0.52, 0.71]
	CE-T1 0.60 [0.52, 0.70]				
LRRFS					
C-Index-training	0.68	0.98	0.68	0.98	0.73
	[0.48, 0.71]	[0.98, 1.00]	[0.65, 0.91]	[0.98, 1.00]	[0.66, 0.89]
C-Index-validation	ADC 0.64 [0.51, 0.76]	0.43 [0.25, 0.59]	0.52 [0.4, 0.62]	**0**.**42** [0.24, 0.58]	**0**.**59** [0.48, 0.70]
	CE-T1 0.64 [0.53,0.75]				
CSM					
C-Index-training	0.66	0.85	0.84	0.85	0.85
	[0.59, 0.80]	[0.77, 0.91]	[0.76, 0.90]	[0.75, 0.90]	[0.73, 0.86]
C-Index-validation	ADC 0.63 [0.49, 0.74]	0.63 [0.49, 0.76]	0.61 [0.49, 0.72]	**0**.**65** [0.50, 0.77]	**0**.**64** [0.52, 0.75]
	CE-T1 0.60 [0.48, 0.70]				

Bold values indicate C-index clinical-radiomic models in the validation group

ADC, apparent diffusion coefficient map; CE-T1, contrast enhancement T1 sequence; OS, overall survival; LRRFS, locoregional recurrence-free survival; CSM, cause-specific mortality

^1^For validation, two C-indexes were reported on the basis of the patients included in the ADC (*N* = 83) or CE-T1(*N* = 108) analysis

**Table 3:** Posttreatment model C-index in the training and in the validation group

Posttreatment model			
Sequence	Clinical^1^	Clinical-Radiomic	
		ADC	CE-T1
OS			
C-Index-training	0.82 [0.74, 0.86]	0.84 [0.79, 0.92]	0.86 [0.80, 0.91
C-Index-validation	ADC 0.68 [0.57, 0.78]	**0**.**69** [0.59, 0.78]	**0**.**65** [0.56, 0.73]
	CE-T1 0.70 [0.6, 0.78]		
LRRFS			
C-Index-training	0.77 [0.67, 0.85]	0.98 [0.98, 1.00]	0.83 [0.89, 1.00]
C-Index-validation	ADC 0.67 [0.51, 0.81]	**0**.**44** [0.25, 0.61]	**0**.**69** [0.57, 0.8]
	CE-T1 0.70 [0.58, 0.81]		
CSM			
C-Index-training	0.77 [0.70, 0.86]	0.86 [0.79, 0.91]	0.87 [0.79, 0.91]
C-Index-validation	ADC 0.73 [0.63, 0.84]	**0**.**75** [0.60, 0.84]	**0**.**70** [0.58, 0.79]
	CE-T1 0.74 [0.63, 0.81]		

Bold values indicate C-index clinical-radiomic models in the validation group

ADC, apparent diffusion coefficient map; CE-T1, contrast enhancement T1 sequence; OS, overall survival; LRRFS, locoregional recurrence-free survival; CSM, cause-specific mortality

^1^For validation, two C-indexes were reported on the basis of the patients included in the ADC (*N* = 83) or CE-T1(*N* = 108) analysis

Table 3 shows the comparison between the C-Index in the TG and the fitted C-Index (C-Index–VG) in ADC and CE-T1 sequences for the Model using posttreatment information. The C-Index was generally > 0.5 in VG: OS C-index for radiomic score were 0.59; LRRFS C-index for ADC and CE-T1were 0.43 and 0.52, respectively; CSM C-index for ADC and CE-T1 were 0.63 and 0.61, respectively. In Supplementary Tables 1 and 2 are reported the differences in radiomic features between VG and TG in CE-T1 and ADC sequences, respectively.

The C-indexes for the hybrid clinical-radiomic models in the validation cohort were lower than those in the training cohort but remained > 0.5 in most cases. CE-T1 sequence provided the best fit to the models: the C-indexes obtained were 0.61, 0.59, 0.64 (pretreatment model) and 0.65, 0.69, 0.70 (posttreatment model) for OS, LRRFS and CSM, respectively.

Other additional details are reported in the Supplementary Material (S1–S2).

## Discussion

The application of radiomics in HN cancers is a barely explored field, especially when tongue tumors are considered [23]. To date, upon a non-systematic search in Pubmed and Embase with the keywords “radiomic” AND “tongue cancer,” few publications emerge: 11 on Pubmed and 15 on Embase. Of these, 9 are in common between the two search engines, one paper is written in Chinese, 3 are congress abstracts, one is focused on lung cancer and the latter is a review [24–29]. Of the remaining publications, one is a review, and five proposed radiomic models to predict the lymph node status in the neck, occult metastases or lymph node ECE [11, 30–34]. Two articles focused on predicting prognosis through MRI radiomic-based features, and others on tumor grading radiomic determination before histology [10, 35–37].

Regarding the use of MRI, the most recent article by Corti et al. reported how MRI-based radiomic signature could be a prognostic marker for OS in oral cavity cancer patients, comparing it with gene expression signatures [38].

Our manuscript presented the validation of clinical-radiomic models for prognosis prediction in mobile tongue tumors, considering OS, CSM and LRRFS.

The validation phase is crucial for radiomics application in clinical practice. As expected, the C-indexes for the clinical-radiomic models in the VG were lower than those in the TG. This is a common feature in validating studies mainly due to the unavoidable differences between the training and validating cohorts [39]. In the present analysis, the VG resulted to have some worse prognostic histological characteristics compared to the TG. However, the C-indexes still remained > 0.5 in most cases, predicting a good adherence of the hybrid model to reality.

In our previous publication, the combined clinical-radiomic models for prognosis prediction showed a strong association between clinical variables, radiomic features and oncological outcomes in OTSCC [10]. We focused our radiomic study on this specific patient group because it is known that HN cancers are very heterogeneous due to different risk factors, anatomical site and prognosis [40]. This heterogeneity is also reported within the same anatomical site: for example, oral cavity subsites are considered cheek, floor of the mouth, mobile tongue, maxillary tuber, mandible. Tumors of these subsites have different survival rates even within the same staging [40]. Furthermore, the gold standard preoperative imaging also varies by subsites: MRI for OTSCC or cheek; computed tomography for maxillary tuber and mandible. For these reasons and to maximize the models’ accuracy, the study group was selected as OTSCC patients and not general oral cavity cancer.

As already mentioned, MRI is the gold standard imaging for local evaluation in OTSCC [7]. The protocol for proper MRI in these patients includes T1- and T2-weighted sequences, CE-T1 with fat saturation and optionally DWI and ADC. MRI can add important information such as preoperative radiological DOI which has been demonstrated to be an independent preoperative predictor of oncological outcomes in OTSCC to and to better predict patients’ clinical stage [41]. This is in accordance with the idea that applying radiomics on MRI could represent an added value in prognosis prediction for OTSCC. In this study we selected only CE-T1 sequences and ADC maps because they represented respectively the most widespread and reproducible sequence (CE-T1) and the best fit (ADC) in the previous published models that we aim to externally validate [10].

Our data confirmed that among the clinical models for prognosis prediction, the models with posttreatment information showed on average better performance in oncological outcomes also in the combined clinical-radiomic models.

Notably, upon comparing the radiomic model (radiomic score) alone with the clinical model based on pretreatment information, we found an improvement in terms of C-index in the TG radiomic model. In the VG, the C-indexes values of the two models were very similar, with an average variation of 0.02, 0.001, suggesting that the radiomic score may have been specific to the population in which it was constructed (TG). The radiomic sequences studied and chosen in the TG for constructing the radiomic score were built with a brush on the TG itself. So, the radiomic score could overfit the TG but be less effective in predicting clinical outcomes in the VG.

Moreover, in the VG, all the MRIs studied belong to different hospitals, while the TG features were acquired on MRI performed at the same hospital (IEO) and some differences may be attributable to the differences between the two cohorts.

In the VG, comparing the clinical model built on posttreatment information and the radiomic model alone, we did not find a relevant advantage in the prognosis prediction accuracy of the radiomic model alone.

Focusing on the combined pre and posttreatment clinical-radiomic models in OS, LRRFS and CSM, we presented comparable C-indexes between groups without significant increases.

Our data underlined that the CE-T1 sequences were suitable for prognosis prediction (C-index > 0.5) in OTSCC. This sequence is also widespread in other hospitals, including non-tertiary centers as it is considered standard-of-care for tongue cancer evaluation, allowing the predictive model to be reproduced for all patients and across different facilities [10, 38]. Conversely, DWI sequences and ADC map are not performed in the clinical routine of all centers, thus limiting its potential application [10, 38].

This validation step on the previously created models underlined two different aspects. Firstly, it confirmed the potential ability of radiomics associated with clinical information to predict the prognosis of a single patient, even in heterogeneous cohorts. Secondly, it also highlighted the fragility of the technique due to the critical issues of validation, which is fundamental for models’ exportation and potential applicability to daily clinical practice [41]. The fact that the C-indexes of the VG were good but all lower than those obtained in the TG draws attention to how it could be difficult to speak a common language in this field [42, 43]. The discrepancy in predictive performance between the training and validation sets questions whether overfitting is an issue or if it is due to differences in external validation images.

Moreover, in our study not all patients presented both CE-T1 and ADC MRI sequences, after multidisciplinary and internal discussion we included all patients with CE-T1 data (108 patients), as represents the most widespread and reproducible MRI sequences: its greater number in the sample, with respect to ADC (83 patients), also reflects its potential greater external applicability.

Multiple variables can influence MRI-based-radiomics on OTSCC, including the different machines, ROIs manual segmentation, relatively small number of cohorts analyzed for oral cancers [43, 44]. These aspects partly explain why radiomics represents a still-developing discipline and in its beginning in HN cancers, especially in tongue cancer [13, 43, 45]. Also, the importance of comparing data across study methodologies and structure with other cancer types, in which radiomics is already at a more advanced state, is essential [43]. Our study is one of the few currently published with the intent to associate MRI radiomics with the ability to predict patients’ prognosis in OTSCC, and it presents strengths and limitations. The validation group, different from the TG cohort, should be considered the main strength of our work [46], followed by the completeness of all clinical and follow-up information of all patients. The major limitation is the suboptimal number of patients in the two cohorts. A more significant number of patients and prospective multicentric studies could increase the significance of these results, their validation and reproducibility. This paper focused on the quantitative data demonstrating the significance of the radiomic data alone thus, we did not measure the ADC value or included radiological information on surrounding structures invasion even if they could be important for tumor aggressiveness and prognosis prediction. Moreover, using a multi-parametric image fusion could lead to better results in terms of models’ performance, this could be the subject of a future work on the same expanded dataset.

## Conclusion

Radiomics represents a promising noninvasive method for the implementation of precision medicine. Our results confirmed that the validation step is one of the main limitations in radiomics, which is more evident when external cohorts are at stake. Nevertheless, our results confirmed the potential added value of radiomics to refine individual patients’ prognosis, even in the context of rather heterogeneous populations. The challenges in external validation may derive from many reasons, including different types of imaging acquisition parameters and variability of tumor volume delineation. Upon solving these issues, radiomics could be a potential instrument to perform tailored prognosis prediction in OTSCC.

## Supplementary Information

Below is the link to the electronic supplementary material.

Supplementary file1 (DOCX 32 KB)

Supplementary file2 (XLSX 33 KB)
