# Supplementary material for: The prognostic role of MRI-based radiomics in tongue carcinoma: a multicentric validation study
Source: Radiol Med. 2024 Aug 3;129(9):1369–81. doi: 10.1007/s11547-024-01859-y (PMC11379741; doi:10.1007/s11547-024-01859-y)
Supplement: Supplementary file 1 — Supplementary file1 (DOCX 32 KB) [file 11547_2024_1859_MOESM1_ESM.docx]

**SUPPLEMENTARY MATERIAL (S1, S2)**

**S1. PRE-TREATMENT MODEL VALIDATION**

**S1.a Overall Survival**

In the previously published pre-treatment clinical model, sex, age, and stage were significantly associated with OS (C-index=0.70)^10^. When this model was applied to the VG, we obtained slightly lower C-indexes: 0.60 for both the subgroups of patients with ADC and CE-T1 (Table 2).

When we fitted a new model to the VG with the same variables selected for the model built in the TG, OS was not different for sex (HR=0.97, p=0.92) and for clinical early stages vs advanced stages (HR=1.69, p=0.32). It was confirmed to be slightly worse for older patients (HR=1.03, p=0.003).

In the previously published pre-treatment clinical-radiomic model, C-indexes were 0.76 and 0.82 for ADC and CE-T1, respectively. When this model was applied to the VG, ADC sequences resulted in C-Index=0.60 (95% CI: 0.49, 0.71), while for CE-T1 sequences, C-Index was 0.61 (95% CI: 0.52, 0.71) (Table 2).

**S1.b Loco Regional Recurrence Free Survival**

In the previously published pre-treatment clinical model, sex and age were not significantly associated with LRRFS. In contrast, cN was positively associated with LRRFS (C-index=0.68)^10^. When this model was applied to the VG, we obtained similar C-indexes: 0.64 for both the subgroup of patients with ADC and CE-T1 (Table 2).

However, when we fitted a new model to the VG with the same variables selected for the model build in the TG, we found different clinical variables associated with LRRFS. Specifically, HR for sex and age were significant (HR=0.39; p=0.039 and HR=1.05; p=0.006, respectively), while cN+ vs cN0 was not anymore significant (HR=1.02; p=0.97).

In the previously published pre-treatment clinical-radiomic model, C-indexes were 0.98 and 0.73 for ADC and CE-T1, respectively. When this model was applied to the VG, ADC sequences resulted in C-Index=0.42, (95% CI: 0.24, 0.58) and CE-T1 sequence C-Index=0.59 (95% CI: 0.48, 0.7) (Table 2).

**S1.c Cause-Specific Mortality**

In the previously published pre-treatment clinical model, sex and age were not significantly associated with CSM, while stages III and IV were positively associated with CSM (C-index=0.66)^10^. When this model was applied to the VG, we obtained similar C-indexes: 0.63 and 0.60 for the subgroup of patients with data on ADC and with data on CE-T1 (Table 2).

However, when we fitted a new model to the VG with the same variables selected for the model build in the TG, we found that only the age at diagnosis was significantly associated with a worse survival outcome (HR=1.03, 95% CI [1.00-1.06] p=0.03).

In the previously published pre-treatment clinical-radiomic model, C-indexes were 0.85 for ADC and CE-T1. When this model was applied to the VG, ADC sequences resulted in C-Index=0.65, (95% CI [0.5, 0.77], while for CE-T1 sequences, C-Index was 0.64 95%CI [0.52,0.75] (Table 2).

**S2. POST-TREATMENT MODEL VALIDATION**

**S2.a Overall Survival**

In the previously published post-treatment clinical model, female sex, older age, and pN+ were significantly associated with a higher risk of death (C-index=0.82)^10^. When this model was applied to the VG, we obtained slightly lower C-indexes: 0.68 and 0.70 for the subgroup of patients with data on ADC and with data on CE-T1, respectively (Table 3).

When we fitted a new model to the VG with the same variables selected for the TG model, OS was confirmed to be significantly associated with age (HR=1.04, p=0.001) and pN+ (HR= 2.49 and 3.20; p=0.018 and 0.002 for pN+ ECE- and pN+ ECE+, respectively), but not with sex (HR=0.97, p=0.93).

In the previously published pre-treatment clinical-radiomic model, C-indexes were 0.84 and 0.86 for ADC and CE-T1, respectively. When this model was applied to the VG, ADC sequences resulted in C-Index=0.69 (95% CI: 0.59, 0.78), while for CE-T1 C-Index was 0.65 (95% CI: 0.56, 0.73) (Table 3).

**S2.b Loco Regional Recurrence Free Survival**

In the previously published post-treatment clinical model, positive surgical margins and pN+ ECE+ were associated with the worst prognosis in terms of LRRFS (C-index=0.77)^10^. When this model was applied to the VG, we obtained slightly lower C-indexes, although a good prediction was confirmed: 0.67 for the subgroup of patients with data on ADC and 0.7 for that with data on CE-T1 (Table 3).

However, when we fit a new model to the VG with the same variables selected for the model build in the TG, we found different clinical variables associated with LRRFS. Specifically, sex and age were significant (HR=0.39, p=0.039 and HR=1.05, p=0.006, respectively), while positive surgical margins and pN+ ECE+ was no more significant (HR=1.98; p=0.15 and 2.12; p=0.20, respectively).

In the previously published post-treatment clinical-radiomic model, C-indexes were 0.98 and 0.83 for ADC and CE-T1, respectively. When this model was applied to the VG, ADC sequences resulted in lower C-Indexes=0.44, (95% CI: 0.25, 0.61), while for CE-T1 sequences, C-Index was 0.69 (95% CI: 0.57, 0.8) (Table 3).

**S2.c Cause-Specific Mortality**

Female sex and pN+ were significantly associated with a worse prognosis in terms of CSM (C-index=0.77), in the previously published post-treatment clinical model^10^. When this model was applied to the VG, we obtained similar C-indexes: 0.73 and 0.74 for ADC and CE-T1, respectively (Table 3).

When we fitted a new model to the VG with the same variables selected for the model built in the TG, we confirmed the worst prognosis for patients with pN+ and we found a significant association with CSM for age (HR=4.79, p=0.008; HR=8.38, p<0.0001; HR= 1.03, p=0.012, respectively for pN+ ECE-, pN+ ECE+ and age).

In the published post-treatment clinical-radiomic model, C-indexes were 0.86 and 0.87 for ADC and CE-T1, respectively^10^. When this model was applied to the VG, ADC sequences resulted in still higher C-Indexes=0.75, (95% CI: 0.60, 0.84) and CE-T1 sequences C-Index was 0.70 (95% CI: 0.58, 0.79) (Table 3).

**SUPPLEMENTARY TABLES**

**Supplementary Table 1:** **Differences in radiomic features between Validation and Training Groups in CE-T1 sequences**

| CE-T1 characteristics | VG: BS  N = 64^1^ | VG: IEO EXT  N = 25^1^ | VG: PV  N = 19^1^ | TG  N = 79^1^ | P-VALUE^2^ | | P-VALUE ADJUSTED |
| --- | --- | --- | --- | --- | --- | --- | --- |
| Exponential_glszm_smallarealowgraylevelemphasis | 0.05 (0.04, 0.08) | 0.04 (0.01, 0.05) | 0.06 (0.03, 0.11) | 0.11 (0.07, 0.15) | | <0.001 | 0.000 |
| Lbp-3d-m2_firstorder_rootmeansquared | 11.82 (11.68, 11.99) | 12.84 (11.99, 13.41) | 12.08 (11.77, 13.04) | 11.77 (11.59, 12.18) | | <0.001 | 0.000 |
| Lbp-3d-k_glszm_zoneentropy | 2.50 (2.32, 2.64) | 2.40 (2.13, 2.79) | 2.40 (1.99, 2.59) | 2.37 (2.01, 2.53) | | 0.032 | 0.058 |
| Log-sigma-1-0-mm-3d_glcm_mcc | 0.72 (0.68, 0.74) | 0.58 (0.44, 0.74) | 0.67 (0.48, 0.69) | 0.73 (0.56, 0.79) | | 0.002 | 0.006 |
| Log-sigma-2-0-mm-3d_glszm_sizezonenonuniformity | 11 (5, 24) | 20 (5, 101) | 22 (5, 42) | 22 (10, 49) | | 0.034 | 0.059 |
| Log-sigma-2-0-mm-3d_glszm_smallarealowgraylevelemphasis | 0.007 (0.005, 0.010) | 0.006 (0.003, 0.011) | 0.010 (0.006, 0.014) | 0.009 (0.006, 0.012) | | 0.012 | 0.025 |
| Log-sigma-5-0-mm 3d_gldm_smalldependencehighgraylevelemphasis | 0.33 (0.22, 0.65) | 0.88 (0.35, 12.41) | 0.71 (0.26, 1.57) | 1.02 (0.40, 1.83) | | <0.001 | 0.000 |
| Log-sigma-5-0-mm-3d_glszm_sizezonenonuniformitynormalized | 0.05 (0.04, 0.07) | 0.13 (0.06, 0.19) | 0.06 (0.06, 0.10) | 0.10 (0.07, 0.13) | | <0.001 | 0.000 |
| Square_glcm_clustershade | 282 (56, 732) | 4 (-21, 210) | 174 (8, 896) | 4 (-50, 123) | | <0.001 | 0.000 |
| Squareroot_gldm_largedependencelowgraylevelemphasis | 0.06 (0.03, 0.19) | 0.20 (0.07, 0.77) | 0.11 (0.02, 0.38) | 0.03 (0.02, 0.07) | | <0.001 | 0.000 |
| Wavelet-lhl_firstorder_maximum | 339 (250, 430) | 148 (77, 249) | 249 (144, 292) | 305 (215, 394) | | <0.001 | 0.000 |
| Wavelet-hhh_gldm_largedependencelowgraylevelemphasis | 10 (5, 19) | 19 (2, 74) | 8 (4, 26) | 7 (3, 11) | | 0.011 | 0.023 |
| CE-T1: Contrast Enhancement T1 sequence. VG: Validation Group. BS: Brescia Spedali Civili. IEO EXT: Patient treated at IEO with MRI performed in other hospitals. PV: Pavia San Matteo. TG: Training Group | | | | | | | |

**Supplementary Table 2: Differences in radiomic features between Validation and Training Groups in ADC sequences**

| ADC characteristics | VG: BS  N = 63^1^ | VG: IEO EXT  N = 13^1^ | VG: PV  N = 7^1^ | TG  N = 79^1^ | P-VALUE^2^ | P-VALUE ADJUSTED |
| --- | --- | --- | --- | --- | --- | --- |
| Wavelet-lhl_firstorder_median | 0.69 (0.65, 0.75) | 0.64 (0.55, 0.66) | 0.60 (0.58, 0.74) | 0.70 (0.65, 0.75) | 0.01 | 0.024 |
| Wavelet-lhh_firstorder_uniformity | 33 (23, 57) | 16 (12, 25) | 35 (26, 70) | 10 (8, 12) | <0.001 | 0.000 |
| Wavelet-lhh_gldm_largedependencelowgraylevelemphasis | 0.67 (0.56, 0.76) | 0.47 (0.40, 0.66) | 0.64 (0.54, 0.72) | 0.32 (0.27, 0.42) | <0.001 | 0.000 |
| Wavelet-hll_firstorder_skewness | 2.33 (2.08, 2.59) | 3.30 (2.39, 4.95) | 2.32 (2.06, 2.70) | 3.09 (2.64, 3.63) | <0.001 | 0.000 |
| Wavelet-hll_glcm_mcc | 22 (19, 25) | 17 (14, 24) | 24 (20, 25) | 17 (13, 21) | <0.001 | 0.000 |
| Wavelet-hll_glszm_largearealowgraylevelemphasis | 2.0 (1.2, 2.8) | 4.7 (1.7, 11.9) | 8.1 (3.3, 9.2) | 2.6 (1.3, 4.1) | 0.002 | 0.007 |
| Wavelet-hlh_glszm_largeareahighgraylevelemphasis | 0 (-31, 29) | -7 (-34, 16) | -2 (-29, 37) | 67 (25, 93) | <0.001 | 0.000 |
| Wavelet-hlh_ngtdm_complexity | 0.19 (0.16, 0.22) | 0.21 (0.19, 0.28) | 0.19 (0.15, 0.21) | 0.24 (0.20, 0.28) | <0.001 | 0.001 |
| Wavelet-hhl_firstorder_kurtosis | 0.31 (0.25, 0.40) | 0.23 (0.16, 0.30) | 0.25 (0.21, 0.31) | 0.34 (0.28, 0.41) | 0.018 | 0.035 |
| Wavelet-hhh_glszm_sizezonenonuniformitynormalized | -0.50 (-2.64, 0.08) | -0.82 (-1.93, 0.13) | -1.07 (-5.04, -0.46) | 0.06 (-0.39, 0.36) | <0.001 | 0.000 |
| Wavelet-lll_glcm_correlation | 1,976 (1,405, 2,503) | 3,003 (2,036, 3,811) | 2,855 (2,363, 3,965) | 2,137 (1,709, 2,665) | 0.015 | 0.032 |
| Wavelet-lll_glcm_mcc | 3.62 (3.15, 4.72) | 4.86 (3.95, 7.72) | 6.03 (4.77, 8.26) | 3.60 (3.19, 4.22) | 0.001 | 0.003 |
| Wavelet-lhl_firstorder_median | 4 (-8, 11) | 0 (-2, 4) | 0 (-2, 4) | 15 (8, 23) | <0.001 | 0.000 |
| Wavelet-lhh_firstorder_uniformity | 0.08 (0.07, 0.10) | 0.12 (0.06, 0.27) | 0.14 (0.10, 0.15) | 0.07 (0.07, 0.09) | 0.018 | 0.035 |
| Wavelet-lhh_gldm_largedependencelowgraylevelemphasis | 0.05 (0.03, 0.09) | 0.08 (0.03, 0.82) | 0.07 (0.04, 0.35) | 0.04 (0.03, 0.06) | 0.014 | 0.031 |
| Wavelet-hll_firstorder_skewness | -0.07 (-0.37, 0.33) | 0.22 (0.09, 0.31) | -0.21 (-0.31, 0.31) | -0.21 (-0.62, 0.00) | 0.002 | 0.005 |
| Wavelet-hll_glcm_mcc | 0.87 (0.73, 0.96) | 0.65 (0.51, 0.79) | 0.74 (0.66, 0.76) | 0.88 (0.71, 0.96) | 0.009 | 0.024 |
| Wavelet-hll_glszm_largearealowgraylevelemphasis | 0.02 (0.01, 0.03) | 0.14 (0.01, 2.33) | 0.05 (0.03, 0.08) | 0.02 (0.01, 0.03) | 0.027 | 0.047 |
| Wavelet-hlh_glszm_largeareahighgraylevelemphasis | 1,513 (940, 2,786) | 7,237 (1,459, 33,060) | 11,500 (1,684, 19,111) | 2,048 (1,216, 3,570) | 0.01 | 0.024 |
| Wavelet-hlh_ngtdm_complexity | 2,079 (1,107, 3,928) | 1,080 (82, 3,857) | 1,265 (480, 3,373) | 3,660 (2,211, 7,434) | <0.001 | 0.002 |
| Wavelet-hhl_firstorder_kurtosis | 3.9 (3.0, 6.2) | 6.2 (4.0, 13.3) | 7.2 (4.0, 19.1) | 3.8 (3.2, 4.9) | 0.024 | 0.044 |
| Wavelet-hhh_glszm_sizezonenonuniformitynormalized | 0.51 (0.47, 0.57) | 0.46 (0.44, 0.57) | 0.47 (0.45, 0.49) | 0.54 (0.51, 0.67) | <0.001 | 0.002 |
| Wavelet-lll_glcm_correlation | 0.48 (0.35, 0.56) | 0.56 (0.38, 0.65) | 0.63 (0.59, 0.73) | 0.40 (0.27, 0.58) | 0.003 | 0.008 |
| Wavelet-lll_glcm_mcc | 0.99 (0.95, 1.00) | 0.94 (0.85, 0.98) | 0.97 (0.93, 0.99) | 0.97 (0.86, 0.99) | 0.054 | 0.091 |
| ADP: Apparent Diffusion Coefficient map. VG: Validation Group. BS: Brescia Spedali Civili. IEO EXT: Patient treated at IEO with MRI performed in other hospitals. PV: Pavia San Matteo. TG: Training Group | | | | | | |
